# Seizure Outcomes of Epilepsy Surgery in Children

**DOI:** 10.15844/pedneurbriefs-35-2

**Published:** 2021-01-06

**Authors:** Hyman G. Frankel, Sandi Lam

**Affiliations:** 1 Division of Pediatric Neurosurgery, Ann & Robert H Lurie Children's Hospital, Chicago, IL; 2 Department of Neurosurgery, Northwestern University Feinberg School of Medicine, Chicago, IL

**Keywords:** Epilepsy, Seizures, Pediatric, Children, Epilepsy Surgery, Surgery

## Abstract

Investigators from the Hospital for Sick Children in Toronto reviewed the literature pertaining to seizure outcomes following epilepsy surgery in the pediatric population.

Investigators from the Hospital for Sick Children in Toronto reviewed the literature pertaining to seizure outcomes following epilepsy surgery in the pediatric population [1]. The aim of this systematic review and meta-analysis of 258 studies was to estimate the effect of surgery on long-term seizure outcomes as compared to medical management; the authors further sub-divided outcomes based on location, pathology, lesional vs non-lesional and incomplete vs complete resection. Eleven studies compared surgery with a medical control group; the odds of seizure freedom were significantly higher in the surgical group compared with medical therapy. Evaluation of 68 studies which included mixed pathologies and surgical locations demonstrated an overall seizure freedom rate of 64.8% at one year, decreasing over the first five years, and precipitously dropping off at ten years. Hemispheric surgery demonstrated the highest seizure freedom rate (74.7%), followed by temporal lobe epilepsy surgery (73.3%) and extratemporal lobe epilepsy surgery (60.2%). Non-lesional epilepsy carried a seizure freedom rate of 51.5% following surgery. Ten studies compared non-lesional and lesional epilepsy which demonstrated a lower odds of seizure freedom in non-lesional epilepsy. Fifteen studies reported outcomes of incomplete versus complete resection with lower odds of seizure freedom following incomplete seizure resection for incomplete resection. The authors conclude that surgery should be the recommended treatment for pediatric patients with drug resistant epilepsy who are eligible for surgery and in particular those who have lesional epilepsy.

COMMENTARY. The global burden of epilepsy in the pediatric population remains substantial, with a prevalence of up to 1%, and 20-30% of those patients further diagnosed with drug resistant epilepsy [2]. Epilepsy brings with it a significant increased risk of developmental and cognitive delay and decreased quality of life for both the patient and their caregivers. Early surgical intervention has shown benefit for cognitive outcomes and quality of life for both the patient and family [3-5]. Despite this evidence, there is often a delay from time of diagnosis to surgical evaluation, and surgery remains an underutilized resource [6-7]. This systematic review highlights significant evidence to the growing cohort of literature supporting the benefits of pediatric epilepsy surgery. This is especially true for patients with lesional epilepsy and should serve as further impetus to refer pediatric patients for surgical evaluation earlier.

## Disclosures

The authors have declared that no competing interests exist.
